# Low Serum LDL-C Is Associated with Poorer Overall Survival and Lipid-Related Molecular Features in Colorectal Cancer

**DOI:** 10.3390/biomedicines14091911

**Published:** 2026-08-26

**Authors:** Juan Wang, Yifang Hsieh, Wenhong Wang, Jing Xu

**Affiliations:** 1 Department of General Surgery, Tianjin Union Medical Center, The First Affiliated Hospital of Nankai University, Nankai University, Tianjin 300121, China; 5020250038@nankai.edu.cn; 2 School of Medicine, Nankai University, Tianjin 300071, China; nickyhsieh0927@gmail.com; 3 Department of Preventive and Health Care, Tianjin Union Medical Center, The First Affiliated Hospital of Nankai University, Nankai University, Tianjin 300121, China; 4 Department of Radiology, Tianjin Union Medical Center, The First Affiliated Hospital of Nankai University, Nankai University, Tianjin 300121, China

**Keywords:** midkine, colorectal cancer, lipid metabolism, low-density lipoprotein cholesterol, prognosis

## Abstract

**Background/Objectives**: We investigated whether preoperative serum low-density lipoprotein cholesterol (LDL-C) is associated with prognosis and midkine (MK; encoded by MDK)-related molecular features in colorectal cancer (CRC). **Methods**: This exploratory translational study integrated a retrospective cohort of 437 surgically treated patients, CRC-focused public transcriptomic analyses of MDK, independent functional enrichment of STRING-derived MK interactors, and immunohistochemistry in tumors from the lowest and highest LDL-C quartiles (n = 10 per group). Overall survival was analyzed using Cox proportional hazards regression. **Results**: MDK mRNA expression was elevated in CRC and was associated with adverse clinicopathological features. STRING-derived MK interactors showed significant Gene Ontology enrichment for lipoprotein- and low-density lipoprotein-particle receptor-related functions. KEGG cholesterol metabolism showed nominal enrichment but did not remain significant after multiple-testing correction. In the clinical cohort, each 1 mmol/L increase in LDL-C was independently associated with a lower mortality risk (adjusted HR = 0.612, 95% CI, 0.361–0.998; *p* = 0.048). Tumors from the lowest LDL-C quartile showed higher MK and c-Fos expression and lower ABCA1 expression than tumors from the highest quartile. **Conclusions**: Lower preoperative LDL-C was associated with poorer overall survival and with exploratory tissue-level molecular differences in CRC. These independent data layers support an associative, hypothesis-generating framework that requires prospective and mechanistic validation.

## 1. Introduction

Colorectal cancer (CRC) remains one of the most prevalent malignancies worldwide and is a major cause of cancer-related morbidity and mortality. Despite substantial advances in screening, surgical techniques, systemic therapy, and molecular classification, the biological heterogeneity of CRC continues to pose major challenges for prognosis prediction and therapeutic stratification. Increasing evidence suggests that metabolic dysregulation may contribute to tumor initiation, progression, and therapeutic resistance in CRC. Metabolic abnormalities, including dysregulated lipid metabolism, altered glucose utilization, insulin resistance, and obesity-related systemic inflammation, have been increasingly implicated in colorectal carcinogenesis [1,2,3]. Beyond the traditional assessment of body mass index, emerging studies indicate that specific metabolic phenotypes may better reflect the biological context of tumor development. Abnormal lipid profiles, ectopic fat deposition, and glucose metabolic disturbances may contribute to CRC through multiple mechanisms, including chronic low-grade inflammation, oxidative stress, altered adipokine signaling, and reprogramming of intracellular energy pathways [4,5,6]. These metabolic alterations may not only shape the tumor microenvironment but also influence tumor aggressiveness, metastatic potential, and patient outcomes.

Among the molecules potentially linking metabolic disturbances to tumor behavior, midkine (MK, gene symbol: MDK) has attracted increasing attention. MK is a heparin-binding growth factor that is normally expressed at low levels in adult tissues but is frequently upregulated in a wide range of human malignancies. Previous studies have demonstrated that MK is involved in cell proliferation, migration, angiogenesis, anti-apoptotic signaling, and inflammatory regulation [7,8,9,10]. In CRC, elevated MK expression has been associated with tumor progression and poor clinical outcomes. Importantly, accumulating evidence also suggests that MK may participate in metabolic regulation by interacting with pathways related to lipid handling, glucose metabolism, and inflammatory signaling, indicating that its biological role may extend beyond conventional oncogenic activity [11,12]. This possibility is particularly relevant in CRC, a disease increasingly recognized as being closely associated with metabolic remodeling. Tumor cells undergo profound metabolic reprogramming to meet the demands of rapid growth and survival under hostile microenvironmental conditions [13,14]. While glucose metabolism has long been emphasized in cancer biology, lipid metabolism has more recently emerged as another critical determinant of tumor behavior [15]. Enhanced lipogenesis, altered cholesterol transport, fatty acid oxidation, and crosstalk with obesity-related metabolic signals may all contribute to CRC progression [16,17,18]. However, the upstream mediators that connect these metabolic alterations with malignant phenotypes remain incompletely understood. Whether MK is associated with metabolic dysregulation and disease progression in CRC warrants further investigation. Throughout this manuscript, MDK refers to the gene or its mRNA expression, whereas MK refers to the encoded protein.

At present, studies focusing on MK in CRC have primarily emphasized its proliferative and pro-invasive effects, whereas its association with metabolic features has been less clearly defined. In particular, the potential relationship between MK and metabolic pathways involving lipid transport, glucose utilization, and downstream metabolic transcriptional regulators remains insufficiently explored. A better understanding of this molecular context may provide new insight into the molecular basis of CRC progression and help identify novel biomarkers or therapeutic targets, especially in metabolically vulnerable patient subgroups.

In the present study, we designed an exploratory translational biomarker analysis to investigate whether systemic lipid status is associated with MDK/MK-related molecular features and prognosis in CRC. The study logic was organized as a stepwise framework: clinical evaluation of LDL-C and survival, CRC-focused public transcriptomic analyses of MDK, an independent STRING-derived MK protein-interaction analysis with GO/KEGG enrichment, literature-guided selection of lipid/cholesterol-related candidate molecules, and exploratory tissue-level assessment in LDL-C-stratified tumors.

## 2. Materials and Methods

### 2.1. Data Acquisition and Preprocessing

Transcriptomic data for CRC were obtained from The Cancer Genome Atlas (TCGA) database via the Genomic Data Commons (GDC) portal. CRC-focused analyses used TCGA-COAD and TCGA-READ data, whereas exploratory cross-cancer expression analyses used TCGA pan-cancer data. The downloaded TCGA expression matrices comprised 480 COAD tumor samples and 41 solid-tissue normal samples and 167 READ tumor samples and 10 solid-tissue normal samples; paired analyses used 41 COAD matched tumor–normal pairs and 10 READ matched tumor–normal pairs. RNA sequencing data in FPKM format were converted to transcripts per million (TPM) and log2-transformed [log2(TPM + 1)] before downstream analyses. For tumor-versus-normal diagnostic analyses, normalized RNA-seq data from TCGA and Genotype-Tissue Expression (GTEx) were obtained from the UCSC Xena platform. STRING was used to construct an MK-centered protein–protein interaction (PPI) context, whereas Kaplan–Meier Plotter was used for public survival analyses based on MDK mRNA expression. Only samples with available expression data and relevant clinical annotation were included in the corresponding analyses; samples lacking key variables required for a specific analysis were excluded from that analysis. The institutional clinical cohort and its nested IHC subset were independent of the public transcriptomic datasets and were used for clinical outcome assessment and exploratory tissue-level assessment, respectively. The overall analytical workflow is summarized in Figure S1.

### 2.2. Differential Expression Analysis

Differential expression of MDK between tumor and normal tissues was evaluated using the Mann–Whitney U test for unpaired samples and the Wilcoxon signed-rank test for paired samples. Differential-expression analyses and related visualizations were performed using R (v4.2.1) and the ggplot2 (v3.4.4).

### 2.3. Survival Analysis

Survival analysis was performed using the Kaplan–Meier Plotter database. Patients were stratified into high- and low-expression groups using the automatically selected optimal cutoff implemented in Kaplan–Meier Plotter. Overall survival (OS) was used as the endpoint. Survival curves were generated using the Kaplan–Meier method and compared using the log-rank test. Hazard ratios (HRs) with 95% confidence intervals (CIs) were calculated when available.

### 2.4. ROC and Clinicopathological Analyses

Receiver operating characteristic (ROC) analysis was conducted using the “pROC” (v1.18.0) in R to evaluate the diagnostic performance of MDK mRNA expression in distinguishing tumor from normal tissues. The area under the curve (AUC) and corresponding 95% CIs were calculated. Associations between MDK mRNA expression and clinicopathological variables, including perineural invasion, lymphatic invasion, pathological N stage, and disease-specific survival (DSS) events, were assessed using the Wilcoxon rank-sum test.

### 2.5. STRING-Derived MK Interaction Analysis and Functional Enrichment

An MK-centered PPI network was generated using the STRING database for Homo sapiens, with experimental evidence selected as the active interaction source, a minimum required interaction score of 0.150, and a maximum of 50 interactors. The resulting 50 STRING-derived MK-interacting proteins were mapped to their corresponding gene symbols for functional enrichment. The full STRING network is shown in Figure S2, and the complete 50-interactor list used for enrichment is provided in Table S1.

Functional enrichment analysis was performed in R version 4.2.1 using clusterProfiler (v4.4.4) with org.Hs.eg.db for human gene annotation and ID conversion. Gene Ontology (GO) enrichment included biological process (BP), cellular component (CC), and molecular function (MF) categories; Kyoto Encyclopedia of Genes and Genomes (KEGG) pathway enrichment was performed separately. All 50 STRING-derived input genes were successfully mapped for GO analysis, whereas 27 mapped genes contributed to the KEGG enrichment denominator. Benjamini–Hochberg-adjusted *p* values were used for multiple-testing correction, and terms with adjusted *p* < 0.05 were considered statistically significant. Lipid/cholesterol-related findings were interpreted as exploratory functional context rather than evidence of a prespecified or causal molecular pathway.

### 2.6. Candidate Molecule Selection

ABCA1 and c-Fos were selected as exploratory, literature-guided candidate molecules after lipid/cholesterol-related functional signals were identified in the STRING-derived MK interaction analysis, particularly the significant lipoprotein-receptor-related GO terms. Candidate selection was based on three explicit exploratory criteria: relevance to lipid or cholesterol homeostasis, a literature-supported relationship with MK-related or AP-1-associated biological signaling, and feasibility of evaluation by immunohistochemistry in formalin-fixed, paraffin-embedded CRC tissues. ABCA1 was selected because of its established role in cholesterol efflux and lipid homeostasis. c-Fos was selected because it is a major component of the AP-1 transcription factor complex and has been implicated in transcriptional programs related to tumor progression and metabolic adaptation [19,20]. These candidates were used to provide biological context for the enrichment findings and to support exploratory tissue-level assessment; they were not considered prespecified mechanistic mediators.

### 2.7. Exploratory Cross-Cancer Contextual Analysis

After ABCA1 and c-Fos had been selected as exploratory, literature-guided candidate molecules, their expression profiles across 33 tumor types were examined using TCGA datasets to provide descriptive cross-cancer context. Expression values were log2-transformed and visualized using radar plots generated in R software version 4.2.1. Tumor-versus-normal differences were assessed using the Wilcoxon rank-sum test. These analyses were performed after candidate selection and were used only to provide descriptive cross-cancer context. The institutional CRC cohort, CRC-focused MDK transcriptomic analyses, STRING-derived MK interaction enrichment analysis, and tissue-level assessment were treated as distinct but complementary evidence layers, whereas the broader cross-cancer expression analyses were retained as exploratory comparative context (Figure S3).

### 2.8. Clinical Cohort and Survival Analysis

A retrospective cohort of 437 patients with histologically confirmed colorectal cancer who underwent surgical resection at Tianjin Union Medical Center between 2020 and 2023 was included in this study. Patients with available preoperative serum low-density lipoprotein cholesterol (LDL-C) measurements, clinicopathological information, and survival follow-up data were included in the analysis. Serum LDL-C was measured as part of routine preoperative biochemical testing before surgery.

For baseline comparisons, patients were categorized according to the cohort median LDL-C level of 2.65 mmol/L. Patients with LDL-C levels below 2.65 mmol/L were classified into the low-LDL-C group (n = 216), whereas those with LDL-C levels of 2.65 mmol/L or higher were classified into the high-LDL-C group (n = 221). In Cox proportional hazards regression analyses, LDL-C was analyzed as a continuous variable, with the hazard ratio representing the change in mortality risk associated with each 1 mmol/L increase in LDL-C.

Overall survival was defined as the interval from the date of surgery to death from any cause or the date of last follow-up. The median follow-up duration was 23 months (interquartile range, 13–42 months; range, 1–58 months), during which 29 deaths were recorded. Patients who remained alive at the last follow-up were censored. The public transcriptomic datasets were independent of the institutional clinical cohort and did not contain matched preoperative serum LDL-C measurements; therefore, patient-level concordance between circulating LDL-C and tumor MDK mRNA expression could not be evaluated in the full cohort.

### 2.9. Immunohistochemistry (IHC)

For exploratory tissue-level assessment, 20 cases were selected from the institutional cohort of 437 surgically treated CRC patients. Patients were classified according to the quartile distribution of preoperative serum LDL-C in the full cohort. The lowest LDL-C quartile was defined as an LDL-C level of 2.15 mmol/L or lower, whereas the highest quartile was defined as an LDL-C level of 3.25 mmol/L or higher. Ten cases were selected from each extreme LDL-C quartile according to the availability of adequate paired tumor and adjacent non-tumor formalin-fixed, paraffin-embedded tissues. Case selection was completed before IHC staining and was independent of the subsequent staining results. This extreme-group sampling strategy was intended to increase biological contrast and support exploratory tissue-level assessment rather than population-level estimation of marker prevalence.

Formalin-fixed, paraffin-embedded (FFPE) tissue samples were sectioned at 4 μm thickness. Immunohistochemical staining was performed using the following primary antibodies: rabbit polyclonal anti-Midkine (MK; catalog no. 11009-1-AP; dilution, 1:200), mouse monoclonal anti-c-Fos (clone 1G2C5; catalog no. 66590-1-Ig; dilution, 1:100), and rabbit polyclonal anti-ABCA1 (catalog no. 26564-1-AP; dilution, 1:500), all purchased from Proteintech Group, Wuhan, China. After deparaffinization in xylene (Sinopharm Chemical Reagent Co., Ltd., Shanghai, China; catalog no. 10023418) and rehydration through a graded ethanol series, antigen retrieval was performed in citrate antigen-retrieval buffer (pH 6.0; catalog no. ZLI-9064; ZSGB-BIO, Beijing, China) using microwave heating for three consecutive 5 min cycles at medium, medium–high, and high power. After natural cooling, the sections were washed three times with phosphate-buffered saline (PBS; pH 7.4) for 5 min each. Endogenous peroxidase activity was blocked by incubation with 3% hydrogen peroxide (Sinopharm Chemical Reagent Co., Ltd., Shanghai, China; catalog no. 10011218) for 25 min at room temperature in the dark. The sections were subsequently blocked with goat serum (catalog no. C0265; Beyotime Biotechnology, Shanghai, China) for 15 min at room temperature and incubated with the respective primary antibodies overnight at 4 °C. After three washes with PBS, the sections were incubated with HRP-conjugated Goat Anti-Mouse IgG (H+L) (Proteintech Group, Wuhan, China; catalog no. SA00001-1) or HRP-conjugated Goat Anti-Rabbit IgG (H+L) (Proteintech Group, Wuhan, China; catalog no. SA00001-2), as appropriate, for 50 min at room temperature. Immunoreactivity was visualized using a 3,3′-diaminobenzidine detection kit (DAB; catalog no. ZLI-9018; ZSGB-BIO, Beijing, China), with color development monitored using an Olympus DP26 digital imaging system (Olympus Corporation, Tokyo, Japan). The sections were counterstained with hematoxylin (Baso Diagnostics Inc., Zhuhai, China; catalog no. BA4041) for 5–10 min, differentiated, blued, dehydrated through graded ethanol, cleared in xylene, and mounted with neutral mounting medium (Solarbio, Beijing, China; catalog no. G8590). Negative-control sections were processed in parallel under identical conditions with the primary antibody omitted. Matched adjacent non-tumor tissues were processed using the same staining protocol and served as within-patient reference tissues. The investigators were blinded to the LDL-C group information during immunohistochemical evaluation. Immunohistochemical staining was manually and semi-quantitatively evaluated based on staining intensity and the percentage of positive cells. Tumor areas suitable for evaluation were assessed while excluding necrotic, hemorrhagic, or non-tumorous regions. Staining intensity was scored as 0, negative; 1, weak; 2, moderate; and 3, strong. The percentage of positive cells was scored as 1, 0–25%; 2, >25–50%; 3, >50–75%; and 4, >75–100%. The final IHC score was calculated by multiplying the intensity score by the positive-cell percentage score, yielding a total score from 0 to 12. All slides were independently scored by both investigators before any consensus discussion.

Discrepancies between the two investigators were resolved by consensus. Comparisons between matched tumor and adjacent non-tumor tissues were performed using the Wilcoxon signed-rank test. Comparisons between the independent lowest- and highest-LDL-C quartile groups were performed using the Mann–Whitney U test.

### 2.10. Statistical Analysis

All statistical analyses were performed using R software version 4.2.1. Continuous variables were compared using the Mann–Whitney U test or Student’s *t*-test, as appropriate. Categorical variables were compared using the chi-square test or Fisher’s exact test. Cox proportional hazards regression was used to estimate hazard ratios and 95% confidence intervals. In the Cox regression models, LDL-C was entered as a continuous variable expressed per 1 mmol/L increase. Variables considered clinically relevant or associated with overall survival in univariable analyses were considered for multivariable modeling. The final multivariable model included age, Charlson comorbidity index, TNM stage, and LDL-C. The proportional hazards assumption was assessed using Schoenfeld residuals, and no significant violations were identified. A two-sided *p* value < 0.05 was considered statistically significant.

### 2.11. Use of AI Tools

Selected non-data-bearing illustrative elements in the graphical abstract were generated using ChatGPT (GPT-5.5 Sol; OpenAI, accessed in July 2026). The tool was not used for study design, data generation, statistical analysis, data interpretation, or formulation of the study conclusions. All AI-assisted graphical elements were reviewed and edited by the authors, who take full responsibility for the final content.

## 3. Results

### 3.1. Prognostic Significance of LDL-C in the Clinical Cohort

Baseline characteristics of the 437-patient clinical cohort are summarized in Table 1. Compared with the high-LDL-C group, patients in the low-LDL-C group had lower HDL-C, total cholesterol, triglyceride, and hemoglobin levels and a higher Charlson comorbidity index. Age, body mass index, TNM stage, tumor location, lymph node involvement, and most other clinicopathological variables did not differ significantly between the groups. These baseline differences suggest that LDL-C strata may also reflect variation in systemic metabolic, nutritional, and comorbidity status.

In univariable Cox regression analysis, higher LDL-C was associated with a lower hazard of death (HR per 1 mmol/L increase = 0.559, 95% CI, 0.335–0.933; *p* = 0.026). Age, Charlson comorbidity index, TNM stage, lymph node involvement, HDL-C, and lymphocyte count were also associated with overall survival in univariable analyses (Table 2).

After adjustment for age, Charlson comorbidity index, and TNM stage, LDL-C remained independently associated with overall survival (adjusted HR per 1 mmol/L increase = 0.612, 95% CI, 0.361–0.998; *p* = 0.048). Thus, lower preoperative LDL-C levels were independently associated with a higher mortality risk in the institutional CRC cohort.

### 3.2. CRC-Focused Relevance of MDK mRNA Expression with Exploratory Cross-Cancer Context

**Figure 1:**
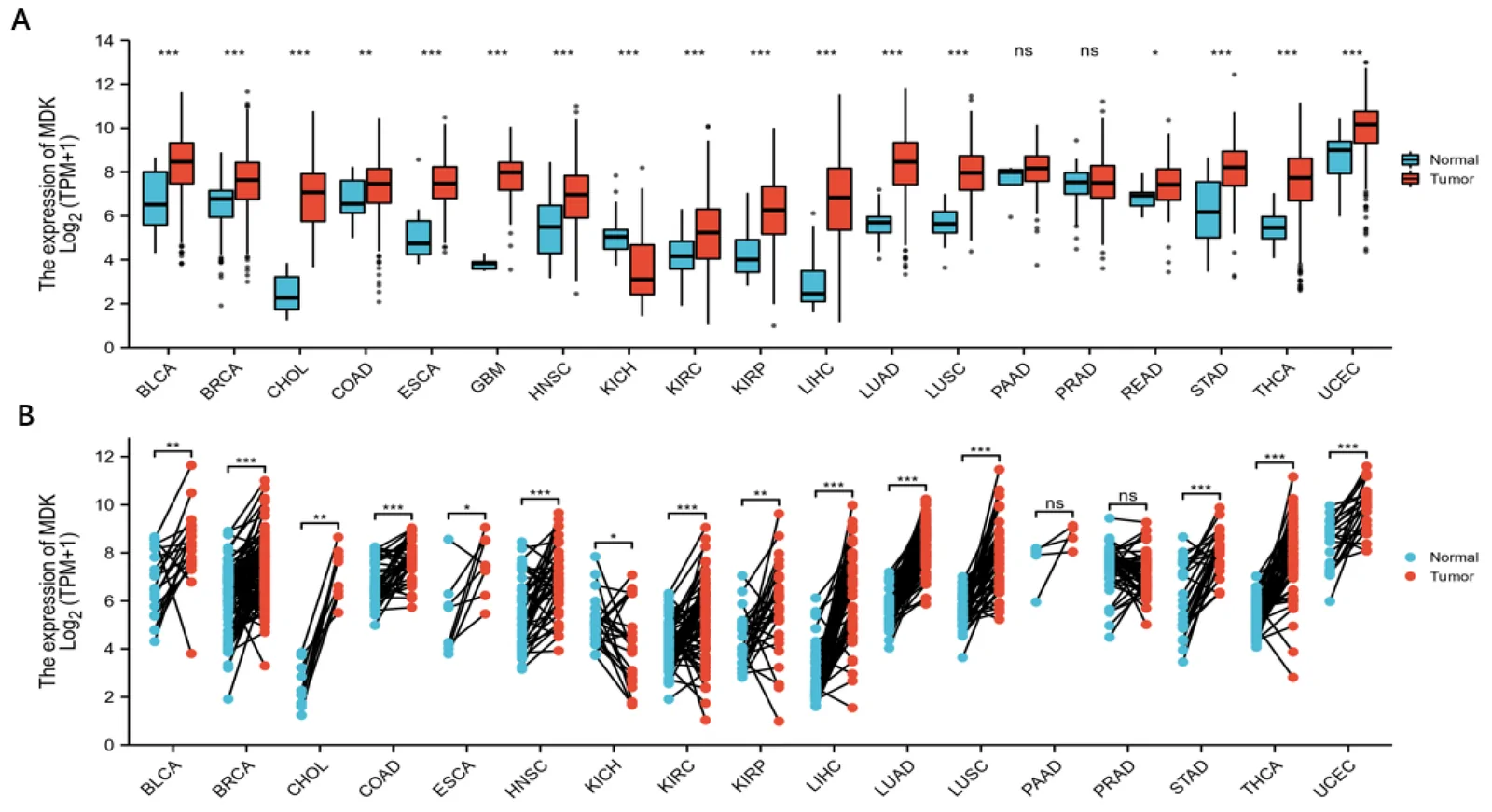
Exploratory cross-cancer expression profile of MDK mRNA: (**A**) MDK mRNA expression in unpaired tumor and normal tissues across multiple cancer types based on TCGA-derived data. (**B**) MDK mRNA expression in paired tumor and adjacent non-tumor tissues where paired samples were available. CRC-related findings contributed to the primary molecular context of the study, whereas findings in other tumor types were retained as exploratory comparative background. Statistical significance was assessed using the Mann–Whitney U test for unpaired comparisons and the Wilcoxon signed-rank test for paired comparisons. * *p* < 0.05, ** *p* < 0.01, *** *p* < 0.001; ns, not significant. Black dots indicate outliers.

CRC-specific findings were considered the primary transcriptomic evidence in the present study, whereas analyses across other tumor types were retained as exploratory comparative context. In the cross-cancer expression analysis, MDK mRNA expression was elevated in tumor tissues relative to normal tissues in multiple cancer types, including the CRC-related COAD and READ datasets (Figure 1A). Paired tumor and adjacent non-tumor analyses showed a broadly similar expression pattern where paired samples were available (Figure 1B). These cross-cancer results provided broader oncological context for the CRC findings.

**Figure 2:**
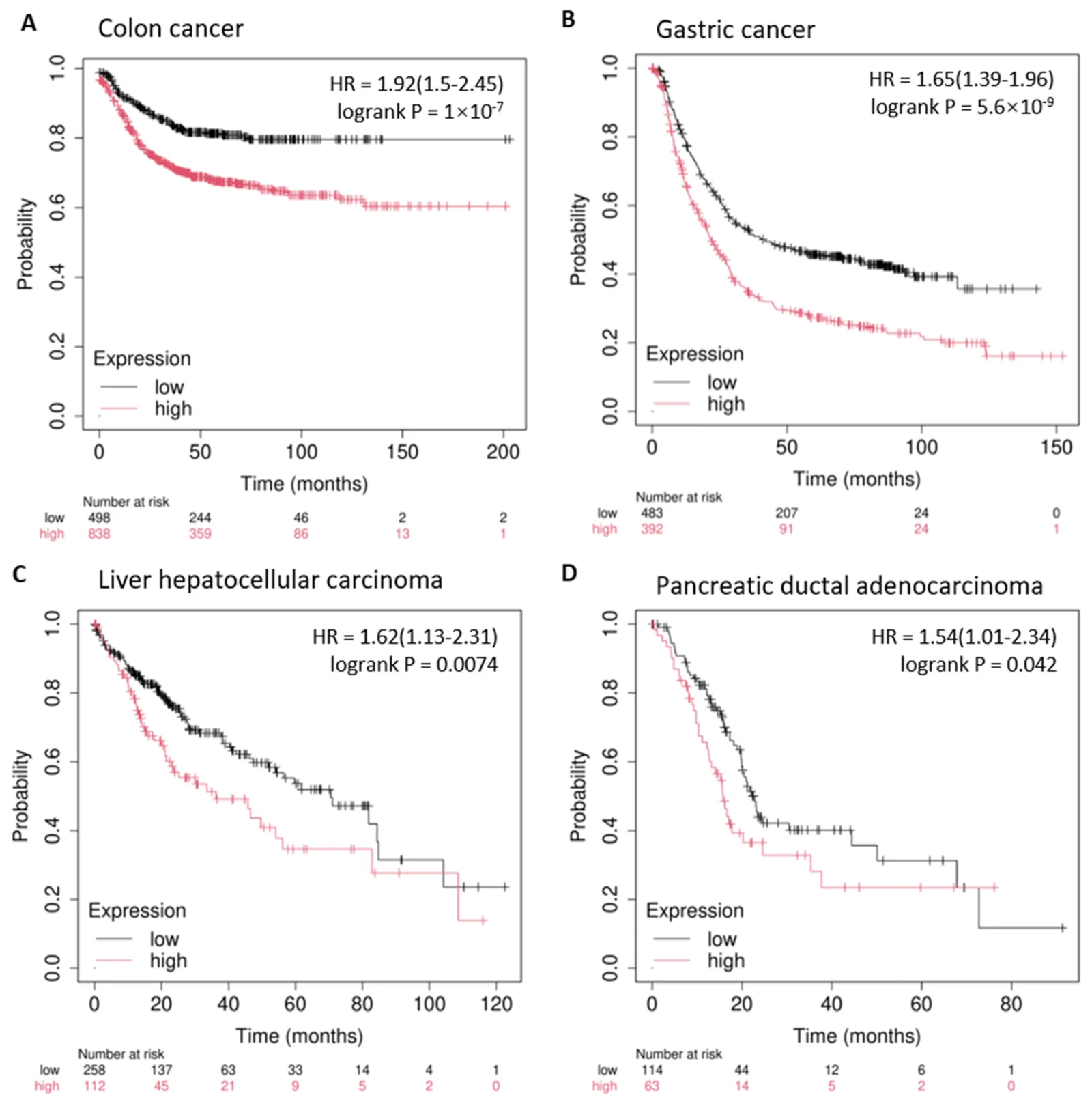
Association between MDK mRNA expression and overall survival in digestive system cancers: (**A**) Colon cancer, representing the CRC-related survival analysis. (**B**) Gastric cancer. (**C**) Hepatocellular carcinoma. (**D**) Pancreatic ductal adenocarcinoma. Panels (**B**–**D**) were included as exploratory comparative context. Overall survival was used as the endpoint, and groups were compared using the log-rank test.

Public survival analysis further showed that higher MDK mRNA expression was associated with poorer overall survival in colon cancer (Figure 2A). Similar associations were observed in gastric, liver, and pancreatic cancers (Figure 2B–D), providing exploratory comparative background.

**Figure 3:**
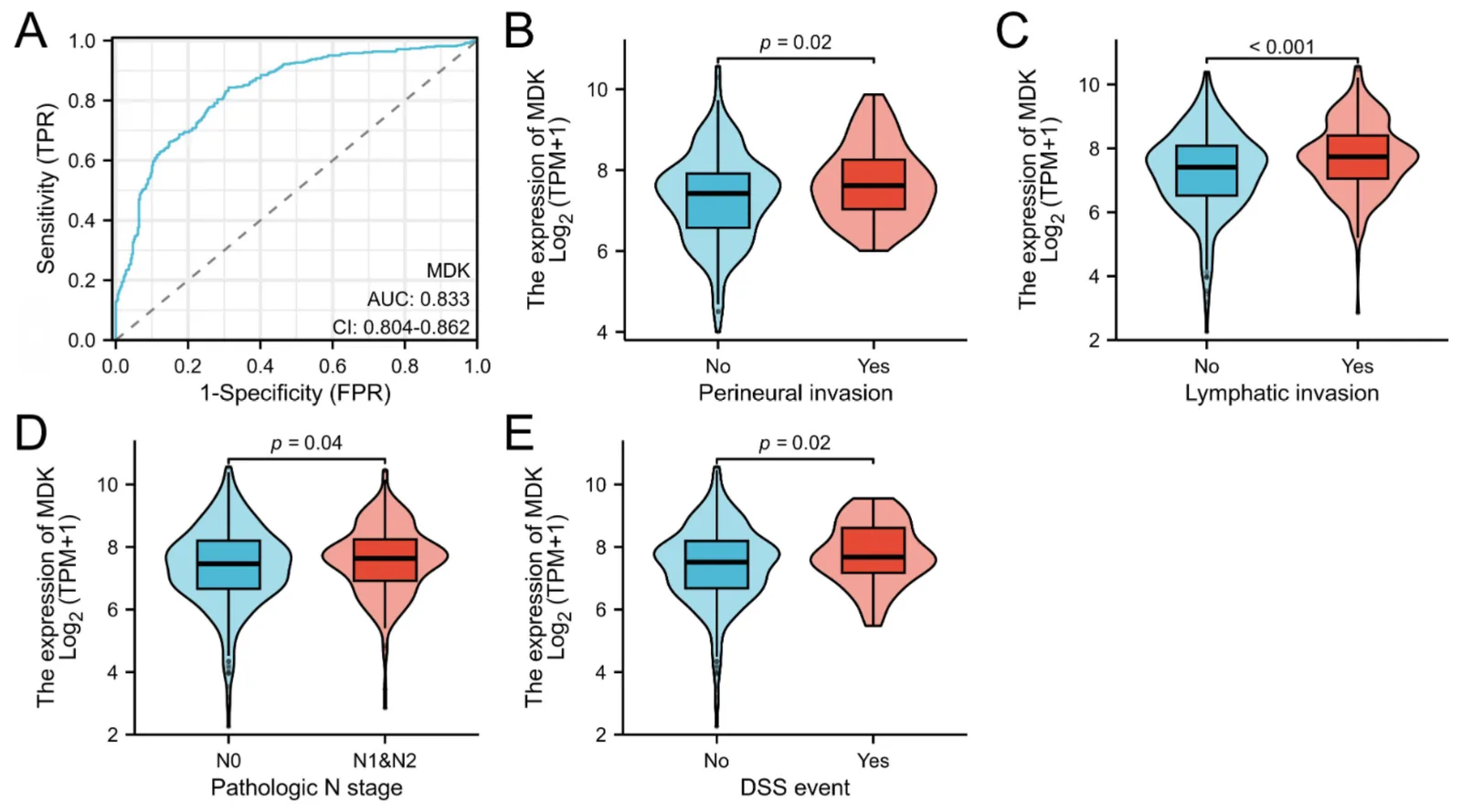
Diagnostic performance and clinicopathological associations of MDK mRNA expression in CRC: (**A**) Receiver operating characteristic curve for distinguishing CRC tissues from normal tissues. Associations between MDK mRNA expression and (**B**) perineural invasion, (**C**) lymphatic invasion, (**D**) pathological N stage, and (**E**) disease-specific survival events. Statistical comparisons were performed using the Wilcoxon rank-sum test. The blue solid line represents the ROC curve, and the dotted diagonal line represents the reference line for no discrimination.

We next evaluated the diagnostic and clinicopathological relevance of MDK mRNA expression using CRC-focused data. MDK mRNA expression discriminated tumor from normal tissues, with an area under the receiver operating characteristic curve of 0.833 (95% CI, 0.804–0.862) (Figure 3A). Higher MDK mRNA expression was also observed in tumors with perineural invasion, lymphatic invasion, advanced pathological N stage, and disease-specific survival events (Figure 3B–E). Collectively, these CRC-specific findings support an association between elevated MDK mRNA expression and adverse clinicopathological characteristics, whereas the broader cross-cancer analyses provide descriptive context only.

### 3.3. STRING-Derived MK Interactors Show Lipid/Cholesterol-Related Functional Enrichment

**Figure 4:**
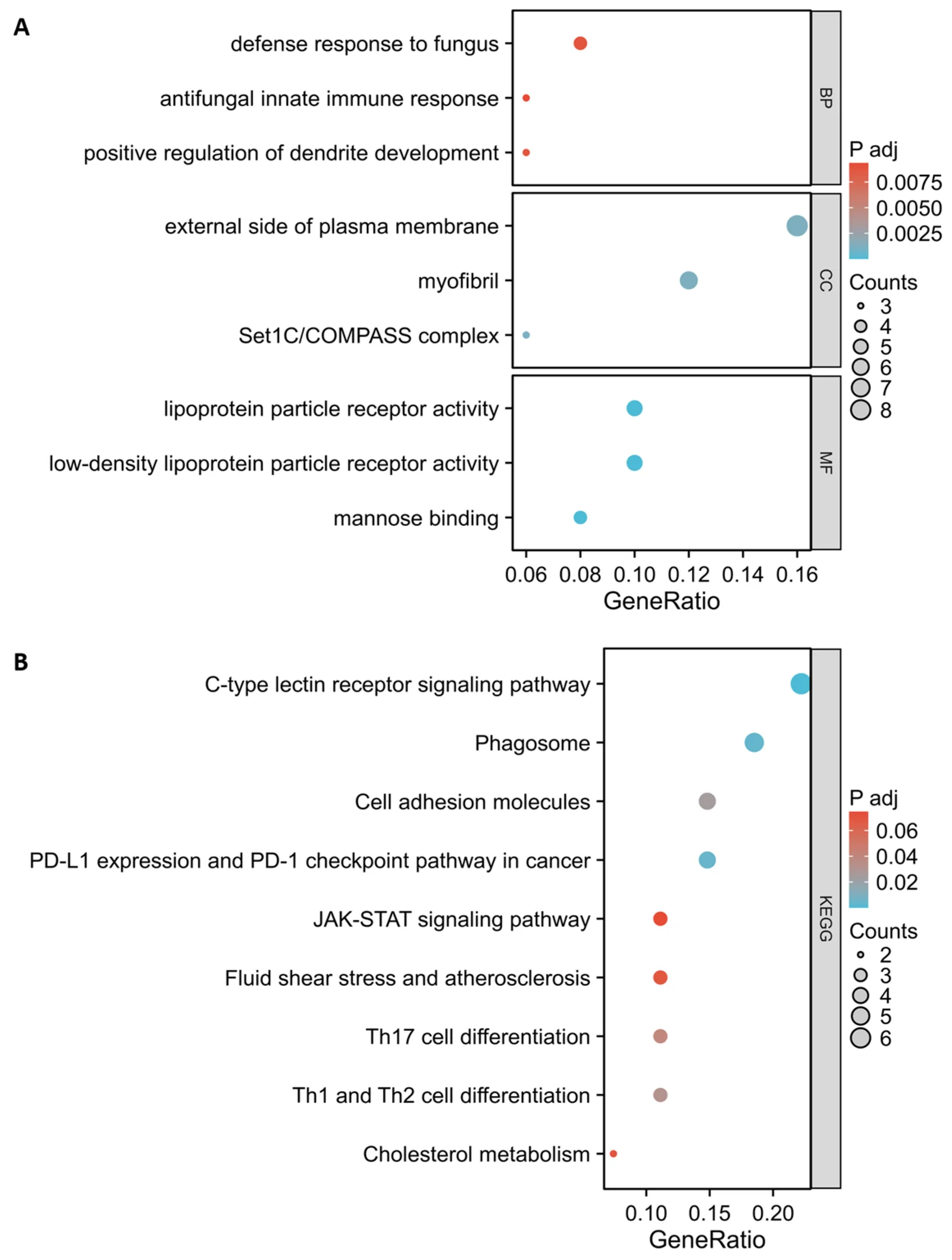
Functional enrichment analysis of STRING-derived MK interactors: (**A**) Selected representative GO biological process (BP), cellular component (CC), and molecular function (MF) terms. Bubble size indicates the number of mapped genes, and color indicates the Benjamini–Hochberg-adjusted *p* value. (**B**) Selected KEGG pathways. Cholesterol metabolism is displayed because of its direct relevance to the study question; it showed nominal enrichment (*p* = 0.0122) but did not remain significant after multiple-testing correction (adjusted *p* = 0.0708).

Independent enrichment analysis of the 50 STRING-derived MK interactors identified significant lipid/cholesterol-related GO signals (Figure 4A). Among the strongest molecular-function terms were low-density lipoprotein particle receptor activity (GO:0005041; 5/50 genes; adjusted *p* = 6.80 × 10^−8^) and lipoprotein particle receptor activity (GO:0030228; 5/50 genes; adjusted *p* = 9.64 × 10^−8^), driven by LRP1, LRP2, LRP6, LRP8, and SORL1. Additional significant terms included apolipoprotein binding (adjusted *p* = 0.0164), lipoprotein particle binding (adjusted *p* = 0.0245), cellular response to cholesterol (adjusted *p* = 0.0327), and cellular response to sterol (adjusted *p* = 0.0347). KEGG analysis identified 14 pathways with adjusted *p* < 0.05. Cholesterol metabolism, involving LRP1 and LRP2, showed nominal enrichment (*p* = 0.0122) but did not remain significant after multiple-testing correction (adjusted *p* = 0.0708) (Figure 4B). Accordingly, the lipid/cholesterol-related findings were interpreted as exploratory functional context rather than evidence of a defined MK-regulated metabolic pathway.

Guided primarily by the significant GO lipid/lipoprotein-related signals and by prior biological evidence, ABCA1 and c-Fos were retained as exploratory, literature-guided candidate molecules. ABCA1 was selected because of its established role in cholesterol efflux and lipid homeostasis, whereas c-Fos was selected because of its involvement in AP-1-related transcriptional programs relevant to tumor progression and metabolic adaptation. Their cross-cancer expression profiles were examined as descriptive comparative context and are presented in Supplementary Figure S3.

### 3.4. Exploratory Tissue-Level Differences in MK, ABCA1, and c-Fos Across LDL-C Strata

**Figure 5:**
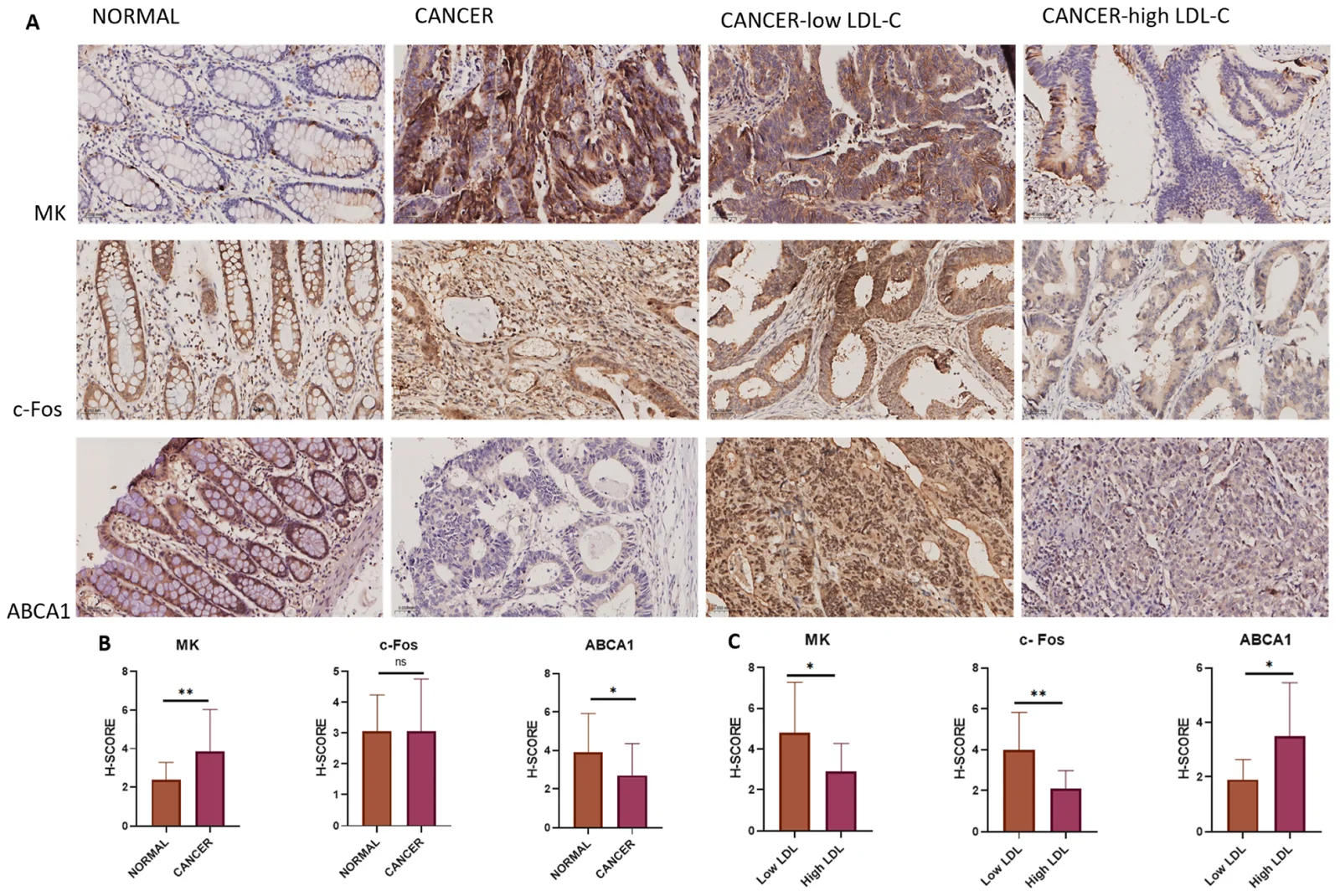
Exploratory immunohistochemical assessment of MK, c-Fos, and ABCA1 expression in CRC tissues: (**A**) Representative immunohistochemical staining of MK, c-Fos, and ABCA1 in CRC tissues and matched adjacent non-tumor tissues, as well as in tumors from patients in the lowest and highest LDL-C quartiles. Scale bar = 50 μm. (**B**) Paired comparisons of MK, c-Fos, and ABCA1 IHC scores between tumor and matched adjacent non-tumor tissues (n = 20 matched pairs). Paired comparisons were performed using the Wilcoxon signed-rank test. (**C**) Comparisons of MK, c-Fos, and ABCA1 IHC scores between tumors from patients in the lowest LDL-C quartile (Q1) and highest LDL-C quartile (Q4) (n = 10 per group). Independent-group comparisons were performed using the Mann–Whitney U test. IHC staining was semi-quantitatively scored by multiplying the staining-intensity score by the positive-cell percentage score. * *p* < 0.05, ** *p* < 0.01; ns, not significant.

To examine whether preoperative LDL-C strata were accompanied by tissue-level molecular differences, we performed an exploratory IHC assessment of MK, c-Fos, and ABCA1 in 20 CRC cases selected from the institutional cohort, including 10 cases from the lowest LDL-C quartile (Q1) and 10 from the highest quartile (Q4). Representative staining patterns are shown in Figure 5A. In paired tumor-versus-adjacent-tissue comparisons, tumor tissues showed higher MK expression and lower ABCA1 expression, whereas c-Fos did not show a consistent paired difference (Figure 5B). In the independent comparison between the extreme LDL-C quartiles, tumors from patients in Q1 showed higher MK and c-Fos expression and lower ABCA1 expression than tumors from patients in Q4 (Figure 5C). Because this assessment included a small number of cases selected from extreme LDL-C strata, the findings were interpreted as exploratory tissue-level associations rather than evidence of a causal or regulatory pathway.

## 4. Discussion

In this exploratory translational study, we investigated whether systemic lipid status was associated with prognosis and tumor-related molecular features in CRC. The principal clinical finding was that lower preoperative LDL-C was independently associated with poorer overall survival in a retrospective cohort of 437 patients. Independent public-dataset analyses provided CRC-focused information on MDK mRNA expression and broader exploratory molecular context, while a separate STRING-derived MK interaction analysis provided protein-interaction-based functional context. Tissue-level assessment further identified differences in MK, ABCA1, and c-Fos expression across extreme LDL-C strata.

The inverse association between preoperative LDL-C and overall survival warrants careful interpretation. In our cohort, lower circulating LDL-C levels were associated with worse OS. Moreover, reduced LDL-C and total cholesterol levels have been reported in treatment-naïve patients with cancer cachexia or pre-cachexia [21]. Rapidly proliferating tumor cells require substantial lipid resources for membrane synthesis, energy production, and signaling, and enhanced lipid uptake and consumption by tumor tissues may contribute to decreased circulating LDL-C levels [22,23,24]. In addition, cancer-associated systemic inflammation and metabolic reprogramming can further disrupt host lipid homeostasis, leading to altered lipid profiles in patients with advanced disease [21,25]. Moreover, low LDL-C levels have been reported to correlate with malnutrition, cancer cachexia, and impaired physiological reserve, all of which are associated with poorer clinical outcomes [26]. In this context, LDL-C may not function as a direct causal factor but rather as a surrogate marker reflecting the complex interaction between tumor metabolic demand and host systemic status. Therefore, the association between low LDL-C and poor prognosis observed in our study is biologically plausible and consistent with the concept that systemic metabolic alterations mirror tumor aggressiveness and host–tumor interactions. This clinical observation provided a clinically relevant context for interpreting the exploratory enrichment findings, although the independent datasets precluded direct patient-level linkage.

To explore the molecular context of this lipid-associated clinical phenotype, we analyzed a STRING-derived MK protein-interaction network. GO enrichment showed robust lipoprotein/LDL-receptor-related molecular functions and significant cellular responses to cholesterol and sterol. In contrast, KEGG cholesterol metabolism showed only nominal enrichment and was not significant after multiple-testing correction. These results therefore support an exploratory lipid/cholesterol-related functional context rather than a defined metabolic pathway. Guided primarily by the significant GO signals and relevant published evidence [19,20], we focused on ABCA1 and c-Fos as exploratory candidate molecules. ABCA1 was biologically relevant because of its role in cholesterol efflux and lipid homeostasis, whereas c-Fos was of interest because of AP-1-related transcriptional programs associated with tumor progression and metabolic adaptation. These markers were not predefined mechanistic targets.

To compare the network-derived functional context with tumor tissue biology, we performed immunohistochemical analysis of MK, ABCA1, and c-Fos in clinical specimens. We observed increased MK expression and reduced ABCA1 expression in tumor tissues, together with distinct c-Fos expression patterns across LDL-C-stratified tumors. Previous studies, primarily in non-cancer or cardiovascular disease contexts, have reported that MK can suppress ABCA1 expression and inhibit cholesterol efflux, while c-Fos has been implicated in transcriptional programs related to tumor progression and metabolic adaptation [19,20]. Whether these regulatory relationships operate in CRC remains unknown. Accordingly, the IHC findings should be viewed as tissue-level associations that are compatible with, but do not validate, the STRING-derived lipid/cholesterol-related functional context.

An exploratory analysis of our clinical cohort showed that lower preoperative LDL-C was independently associated with poorer overall survival, suggesting that systemic lipid status may reflect clinically relevant heterogeneity in colorectal cancer. The clinical association alone, however, could not define the underlying tumor-related molecular context. MK was selected as the molecular focus based on prior evidence linking it to both CRC progression and metabolic regulation, rather than being identified solely from the LDL-C analysis. CRC-focused transcriptomic analyses showed that elevated MDK mRNA expression was associated with adverse clinicopathological characteristics, while the independent STRING PPI analysis identified significant lipid/lipoprotein-related GO functions. Guided by these distinct evidence layers and previous biological evidence, ABCA1 and c-Fos were selected for exploratory tissue-level assessment. The higher MK protein and c-Fos expression and lower ABCA1 expression observed in tumors from patients with low LDL-C provide hypothesis-generating tissue-level context but do not establish a direct LDL-C-MK-ABCA1/c-Fos regulatory axis.

Several limitations should be acknowledged. First, this was a retrospective, single-center study, and residual confounding related to nutritional status, systemic inflammation, lipid-lowering treatment, and cancer therapy cannot be excluded. Second, the number of deaths was limited, which restricted the number of covariates that could be included in the multivariable model and reduced the precision of the survival estimates. Third, matched serum LDL-C and tumor transcriptomic data were unavailable for the full institutional cohort, preventing patient-level evaluation of the relationship between circulating LDL-C and tumor MDK mRNA expression. Fourth, the IHC assessment included only 20 cases selected from extreme LDL-C quartiles; this design increased biological contrast but limited representativeness and statistical precision. Fifth, the STRING-derived PPI enrichment analysis is database-based and not CRC-specific; although it provides an MK-centered functional context, it does not establish pathway activity or causality in CRC. Finally, the observational design and absence of functional experiments preclude causal inference regarding the relationships among LDL-C, MK, c-Fos, and ABCA1.

## 5. Conclusions

This study identified associations among systemic lipid status, MDK/MK-related molecular features, and prognosis in colorectal cancer. Lower preoperative serum LDL-C was independently associated with poorer overall survival. Independent enrichment of STRING-derived MK interactors showed significant lipoprotein/LDL-receptor-related GO functions, while KEGG cholesterol metabolism represented a nominal, non-FDR-significant exploratory signal. Exploratory IHC further showed higher MK and c-Fos expression and lower ABCA1 expression in tumors from patients in the lowest LDL-C quartile than in those from the highest quartile. These findings should be interpreted as associative and hypothesis-generating rather than causal. Larger prospective cohorts with matched serum lipid and tumor molecular data, together with CRC-specific mechanistic experiments, are required to validate this framework and clarify its translational significance.

## Figures and Tables

**Table 1:** Baseline characteristics of patients stratified by LDL-C levels.

Variable	Total (n = 437)	Low LDL-C (n = 216)	High LDL-C (n = 221)	*p* Value
Age, years	64.92 ± 10.62	65.80 ± 10.61	64.05 ± 10.57	0.086
BMI, kg/m^2^	24.19 ± 3.45	24.05 ± 3.45	24.33 ± 3.45	0.392
HDL-C, mmol/L	1.28 ± 0.41	1.23 ± 0.41	1.32 ± 0.41	0.012
Total cholesterol, mmol/L	4.61 ± 1.18	3.81 ± 0.69	5.38 ± 1.03	<0.001
Triglycerides, mmol/L	1.41 ± 0.84	1.30 ± 0.71	1.52 ± 0.94	0.005
Lymphocyte count, ×10^9^/L	1.47 ± 0.57	1.44 ± 0.58	1.50 ± 0.56	0.271
Hemoglobin, g/L	122.57 ± 24.53	117.22 ± 25.96	127.82 ± 21.87	<0.001
NLR	4.11 ± 4.47	4.17 ± 3.98	4.06 ± 4.90	0.800
Albumin, g/L	40.58 ± 5.96	40.07 ± 7.08	41.07 ± 4.57	0.080
Fasting glucose, mmol/L	6.12 ± 1.91	6.17 ± 2.06	6.07 ± 1.76	0.593
CEA, ng/mL	30.38 ± 135.42	33.81 ± 154.54	27.02 ± 113.82	0.603
CCI	5.51 ± 1.87	5.71 ± 1.91	5.30 ± 1.80	0.022
Sex				0.095
Male	265 (60.6%)	140 (64.8%)	125 (56.6%)	
Female	172 (39.4%)	76 (35.2%)	96 (43.4%)	
Smoking status				0.437
Never	256 (58.6%)	128 (59.3%)	128 (57.9%)	
Quit ≥ 2 years	57 (13.0%)	32 (14.8%)	25 (11.3%)	
Light smoker	45 (10.3%)	18 (8.3%)	27 (12.2%)	
Heavy smoker	79 (18.1%)	38 (17.6%)	41 (18.6%)	
Alcohol use				0.078
Never	260 (59.5%)	128 (59.3%)	132 (59.7%)	
Quit ≥ 1 year	47 (10.8%)	30 (13.9%)	17 (7.7%)	
Current	130 (29.7%)	58 (26.9%)	72 (32.6%)	
Diabetes mellitus				0.072
No	367 (84.0%)	174 (80.6%)	193 (87.3%)	
Yes	70 (16.0%)	42 (19.4%)	28 (12.7%)	
Hyperlipidemia				0.628
No	433 (99.1%)	215 (99.5%)	218 (98.6%)	
Yes	4 (0.9%)	1 (0.5%)	3 (1.4%)	
Hypertension				0.498
No	277 (63.4%)	133 (61.6%)	144 (65.2%)	
Yes	160 (36.6%)	83 (38.4%)	77 (34.8%)	
Tumor location				0.320
Rectum	130 (29.7%)	59 (27.3%)	71 (32.1%)	
Colon	307 (70.3%)	157 (72.7%)	150 (67.9%)	
TNM stage				0.894
I	54 (12.4%)	26 (12.0%)	28 (12.7%)	
II	205 (46.9%)	103 (47.7%)	102 (46.2%)	
III	105 (24.0%)	49 (22.7%)	56 (25.3%)	
IV	73 (16.7%)	38 (17.6%)	35 (15.8%)	
Lymph node infiltration				0.577
No	244 (55.8%)	124 (57.4%)	120 (54.3%)	
Yes	193 (44.2%)	92 (42.6%)	101 (45.7%)	

Values are presented as mean ± SD or n (%). Abbreviations: BMI, body mass index; HDL-C, high-density lipoprotein cholesterol; LDL-C, low-density lipoprotein cholesterol; NLR, neutrophil-to-lymphocyte ratio; CCI, Charlson comorbidity index.

**Table 2:** Univariable and multivariable Cox regression analysis for overall survival.

Variable	Univariable HR (95% CI)	*p* Value	Multivariable HR (95% CI)	*p* Value
Age, years	1.058 (1.015–1.104)	0.008	1.052 (1.008–1.099)	0.020
BMI, kg/m^2^	1.042 (0.940–1.156)	0.435		
HDL-C, mmol/L	0.721 (0.521–0.998)	0.048		
Total cholesterol, mmol/L	0.812 (0.655–1.007)	0.059		
Triglycerides, mmol/L	0.903 (0.742–1.099)	0.303		
Lymphocyte count, ×10^9^/L	0.465 (0.219–0.984)	0.045		
Hemoglobin, g/L	0.989 (0.978–1.001)	0.071		
NLR	1.012 (0.968–1.058)	0.602		
Albumin, g/L	0.962 (0.910–1.017)	0.173		
Fasting glucose, mmol/L	1.041 (0.905–1.198)	0.571		
CEA, ng/mL	1.001 (0.999–1.003)	0.220		
CCI	1.478 (1.280–1.708)	<0.001	1.391 (1.188–1.628)	<0.001
Sex	1.263 (0.590–2.702)	0.551		
Smoking status	1.084 (0.879–1.336)	0.445		
Alcohol use	0.892 (0.702–1.132)	0.346		
Diabetes mellitus	1.137 (0.434–2.980)	0.795		
Hyperlipidemia	1.412 (0.318–6.275)	0.653		
Hypertension	0.538 (0.230–1.260)	0.154		
Tumor location	0.738 (0.343–1.590)	0.438		
TNM stage, per stage increment	1.907 (1.277–2.849)	0.002	4.872 (1.902–12.480)	0.001
Lymph node infiltration	2.469 (1.147–5.315)	0.021		
LDL-C, per 1 mmol/L increase	0.559 (0.335–0.933)	0.026	0.612 (0.361–0.998)	0.048

Abbreviations: HR, hazard ratio; CI, confidence interval. Variables with clinical relevance and univariable significance were considered for multivariable analysis. The final model included age, CCI, TNM stage, and LDL-C. LDL-C was modeled as a continuous variable; the HR represents the change in mortality risk associated with each 1 mmol/L increase in LDL-C.

## Data Availability

The public datasets analyzed in this study are available from TCGA/GDC, UCSC Xena, STRING, and Kaplan–Meier Plotter. The complete list of STRING-derived MK interactors used for functional enrichment analysis is provided in Table S1. The clinical cohort data generated and analyzed during the current study are not publicly available due to patient privacy and institutional ethical restrictions, but de-identified data may be available from the corresponding author on request and with approval from the institutional ethics committee.

## Supplementary Materials

The following supporting information can be downloaded at https://www.mdpi.com/article/10.3390/biomedicines14091911/s1, Figure S1: Study workflow; Figure S2: STRING-derived MK-centered protein–protein interaction network; Figure S3: Exploratory cross-cancer expression profiles of FOS and ABCA1; Table S1: Complete list of the 50 STRING-derived MK interactors used for functional enrichment analysis.

## Abbreviations

The following abbreviations are used in this manuscript:

ABCA1	ATP-binding cassette transporter A1
AP-1	Activator protein 1
AUC	Area under the curve
BMI	Body mass index
BP	Biological process
CC	Cellular component
CCI	Charlson comorbidity index
CEA	Carcinoembryonic antigen
CI	Confidence interval
COAD	Colon adenocarcinoma
CRC	Colorectal cancer
DAB	3,3′-diaminobenzidine
DSS	Disease-specific survival
FFPE	Formalin-fixed, paraffin-embedded
FOS	Fos proto-oncogene
GDC	Genomic Data Commons
GO	Gene Ontology
GTEx	Genotype-Tissue Expression
HDL-C	High-density lipoprotein cholesterol
HR	Hazard ratio
IHC	Immunohistochemistry
KEGG	Kyoto Encyclopedia of Genes and Genomes
LDL-C	Low-density lipoprotein cholesterol
MF	Molecular function
MDK	Midkine gene
MK	Midkine protein
NLR	Neutrophil-to-lymphocyte ratio
OS	Overall survival
PPI	Protein–protein interaction
READ	Rectum adenocarcinoma
ROC	Receiver operating characteristic
STRING	Search Tool for the Retrieval of Interacting Genes/Proteins
TCGA	The Cancer Genome Atlas
TPM	Transcripts per million
