# Supplementary material for: Low Serum LDL-C Is Associated with Poorer Overall Survival and Lipid-Related Molecular Features in Colorectal Cancer
Source: Biomedicines. 2026 Aug 26;14(9):1911. doi: 10.3390/biomedicines14091911 (PMC13604130; doi:10.3390/biomedicines14091911)
Supplement: Supplementary file 1 [file biomedicines-14-01911-s001.zip › Supplementary Materials Figure S1.pdf]

Supplementary Materials:

Figure S1: Study workflow.

CRC-focused primary evidence with exploratory molecular context

1. Clinical cohort	2. Public molecular resources	3. MDK-centered analyses	4. MK interaction context	5. Exploratory candidate selection	6. Exploratory tissue-level assessment
437 surgically treated CRC patients<br>Preoperative LDL-C and overall survival<br>Cox regression	TCGA/GTEx via UCSC Xena<br>STRING<br>Kaplan–Meier Plotter<br>Expression, survival, interaction, and enrichment analyses	MDK expression in CRC<br>Survival and diagnostic analyses<br>Clinicopathological associations	STRING-derived MK interactors (n = 50)<br>Experimental evidence; score ≥ 0.150<br>GO and KEGG enrichment<br>Lipid/cholesterol-related functional signals	Lipid/cholesterol-related functional signals + literature evidence<br>ABCA1: cholesterol efflux/lipid homeostasis<br>c-Fos: AP-1-related transcriptional context	IHC in extreme LDL-C strata<br>Lowest (Q1) vs. highest (Q4) LDL-C quartiles<br>n = 10 per group<br>Semi-quantitative scoring

Abbreviations: CRC, colorectal cancer; LDL-C, low-density lipoprotein cholesterol; MK, midkine protein; MDK, the gene encoding midkine; GO, Gene Ontology; PPI, protein–protein interaction; IHC, immunohistochemistry; TCGA, The Cancer Genome Atlas; GDC, Genomic Data Commons; GTEx, Genotype-Tissue Expression.

Figure S1. Study workflow linking the clinical cohort, public molecular resources, CRC-focused MDK analyses, STRING-derived MK-centered interaction analysis, GO/KEGG enrichment, exploratory candidate selection, and tissue-level assessment. CRC-specific analyses constituted the primary evidence, whereas cross-cancer analyses were retained as supportive exploratory context.
