# Supplementary material for: Low Serum LDL-C Is Associated with Poorer Overall Survival and Lipid-Related Molecular Features in Colorectal Cancer
Source: Biomedicines. 2026 Aug 26;14(9):1911. doi: 10.3390/biomedicines14091911 (PMC13604130; doi:10.3390/biomedicines14091911)
Supplement: Supplementary file 1 [file biomedicines-14-01911-s001.zip › Supplementary Materials Figure S2.pdf]

Supplementary Materials:

Figure S2: STRING-derived MK-centered protein–protein interaction network.

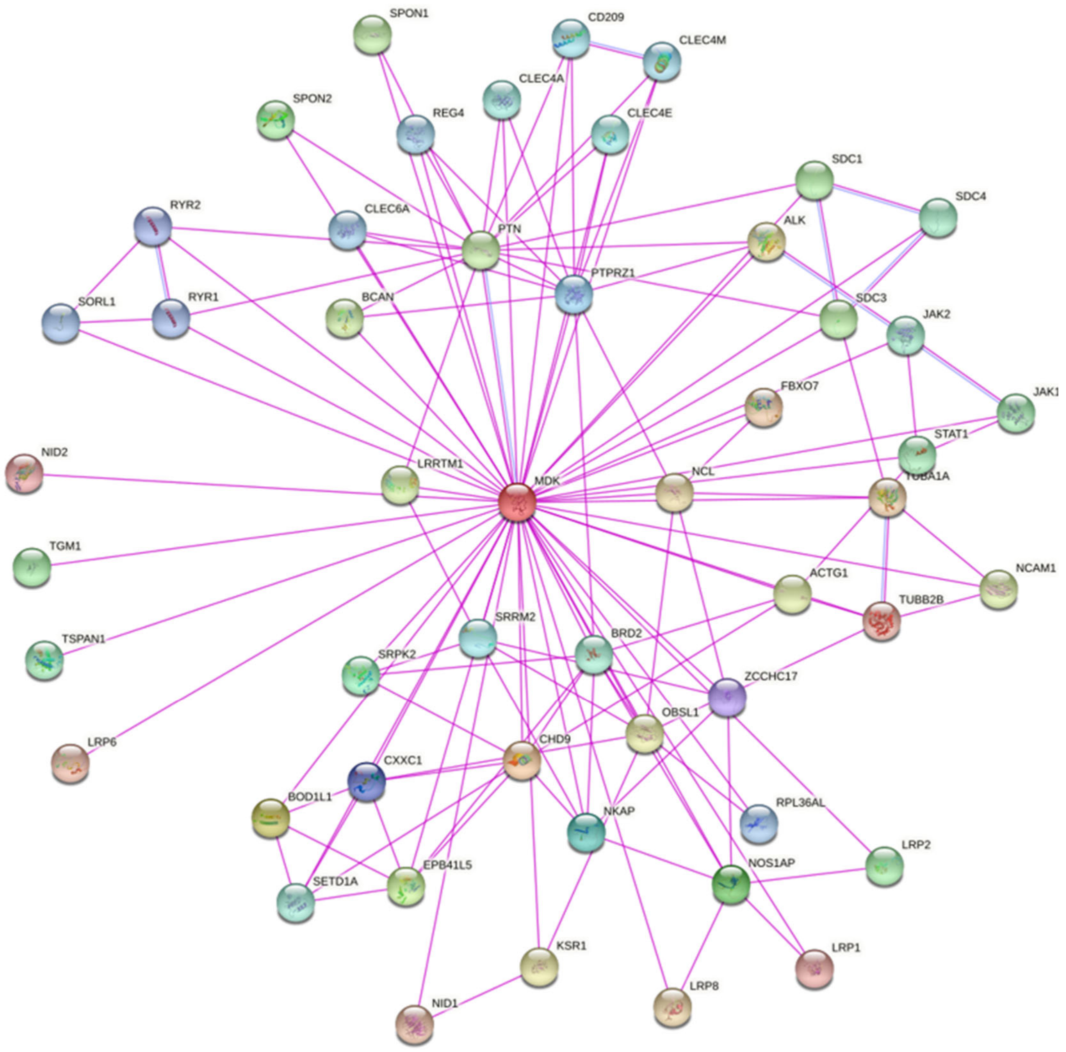

Figure S2. STRING-derived MK-centered protein-protein interaction network used for functional enrichment analysis. The network was generated in STRING for Homo sapiens using experimental evidence as the active interaction source, a minimum required interaction score of 0.150, and a maximum of 50 interactors.
