# Supplementary material for: Low Serum LDL-C Is Associated with Poorer Overall Survival and Lipid-Related Molecular Features in Colorectal Cancer
Source: Biomedicines. 2026 Aug 26;14(9):1911. doi: 10.3390/biomedicines14091911 (PMC13604130; doi:10.3390/biomedicines14091911)
Supplement: Supplementary file 1 [file biomedicines-14-01911-s001.zip › Supplementary Materials Figure S3.pdf]

Supplementary Materials:

Figure S3: Exploratory cross-cancer expression profiles of FOS and ABCA1.

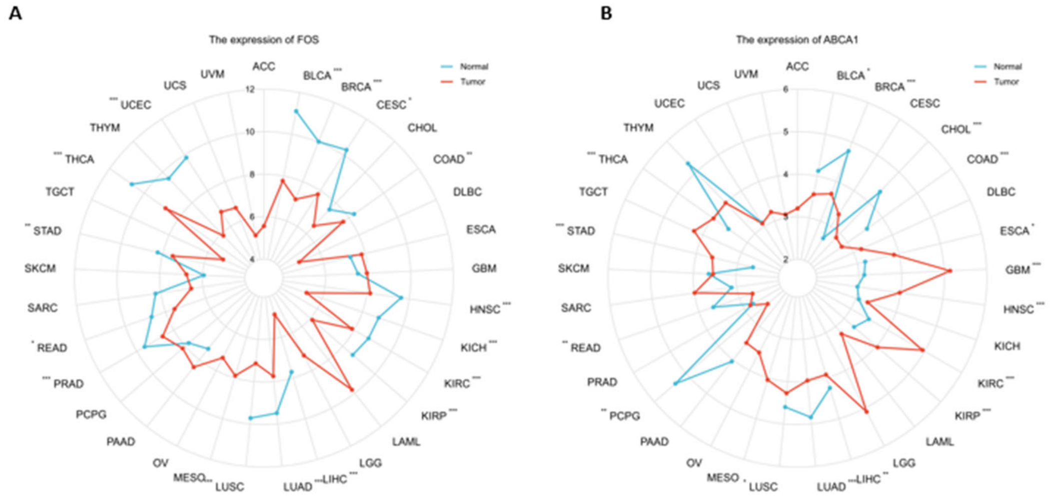

Figure S3. Exploratory cross-cancer expression profiles of FOS (A) and ABCA1 (B) based on TCGA data.
